# Rehabilitation Driven Optimized YOLOv11 Model for Medical X-Ray Fracture Detection

**DOI:** 10.3390/s25185793

**Published:** 2025-09-17

**Authors:** Wenqi Zhang, Shijun Ji

**Affiliations:** 1 School of Nursing, Jilin University, Changchun 130025, China; 2 School of Mechanical and Aerospace Engineering, Jilin University, Changchun 130025, China

**Keywords:** fracture, X-ray, data augmentation, YOLOv11 model, object detection

## Abstract

**Highlights:**

**Main Findings**

- A medical X-ray fracture detection model with precise localization based on YOLOv11n is proposed to solve the problems of false localization and poor accuracy in existing models.
- The improved model, trained with an expanded dataset using data augmentation and enhanced with a Bone-MSCA module and Focal-SIoU loss function, outperforms other mainstream single-object detection models, with significant improvements in detection accuracy, recall rate, F1-Score and mean Average Precision 50.

**Implication of the Main Findings**

- The proposed model can provide more accurate and reliable fracture detection from X-ray images, which is beneficial for timely and appropriate medical treatment.
- The techniques used in this research, such as data augmentation, the Bone-MSCA module and Focal-SIoU loss function, can be a reference for other medical image detection research, potentially improving the performance of related models.

**Abstract:**

Accurately identifying fractures from X-ray images is crucial for timely and appropriate medical treatment. However, existing models suffer from problems of false localization and poor accuracy. Therefore, this research proposes a medical X-ray fracture detection model with precise localization based on the You Only Look Once version 11 nano (YOLOv11n) model. Firstly, a data augmentation technique combining random rotation, translation, flipping and content recognition padding is designed to expand the public dataset, alleviating the overfitting risk due to scarce medical imaging data. Secondly, a Bone-Multi-Scale Convolutional Attention (Bone-MSCA) module, designed by combining multi-directional convolution, deformable convolution, edge enhancement and channel attention, is introduced into the backbone network. It can capture fracture area features, explore multi-scale features and enhance attention to spatial details. Finally, the Focal mechanism is combined with Smoothed Intersection over Union (Focal-SIoU) as the loss function to enhance sensitivity to small fracture areas by adjusting sample weights and optimizing direction perception. Experimental results show that the improved model trained with the expanded dataset outperforms other mainstream single-object detection models. Compared with YOLOv11n, its detection accuracy, recall rate, F1-Score and mean Average Precision 50 increase by 4.33%, 0.92%, 2.52% and 1.24%, respectively, reaching 93.56%, 86.29%, 89.78% and 92.88%. Visualization of the results verifies its high accuracy and positioning ability in medical X-ray fracture detection.

## 1. Introduction

Fracture is a type of bone structure damage caused by trauma or bone diseases, characterized by sudden onset and a high likelihood of triggering complications [1]. As a result of the intensification of global population aging and the increasing frequency of sports-related injuries, the incidence of fractures continues to rise, and consequently, it severely affects the quality of life of patients. The clinical hazards of fractures are not limited to acute injuries; the prevention and treatment of their complications, such as nonunion, infection, and deep vein thrombosis, have become key challenges during the rehabilitation period [2]. Statistics show that approximately 8–10% of fracture patients develop chronic nonunion due to delayed diagnosis or missed diagnosis, requiring secondary surgical treatment, which increases medical costs by 3–5 times. Therefore, early and accurate diagnosis is a core prerequisite for reducing the disability rate of fractures and improving prognosis [3].

Although X-ray imaging remains the first-line method for fracture diagnosis due to its convenience and low cost [4], its clinical application has significant limitations. Firstly, after three-dimensional skeletal structures are projected into two-dimensional images, overlapping anatomical sites (such as ribs and spine), limitations in projection angles, and low contrast of subtle fracture lines (such as crack fractures) result in a missed diagnosis rate of approximately 18–35% [5]. Secondly, in the early stage of callus formation, the small density difference between newly formed bone tissue and soft tissue makes it difficult to distinguish normal healing from delayed healing, and the inability to quantify callus biological activity easily leads to deviations in treatment decisions [6]. Furthermore, variations in operational standards (such as body position and exposure parameters) and physician experience further amplify errors; the misdiagnosis rate of complex fractures in primary hospitals is twice as high as that in tertiary hospitals, and imaging interference from special populations such as children with epiphyses and elderly patients with osteoporosis also increases the difficulty of interpretation. These technical bottlenecks collectively lead to significant challenges in distinguishing fracture areas from normal bone tissue in medical X-ray films, and may even cause over-medical treatment or delayed treatment due to missed diagnoses and misjudgments [7].

Against the backdrop of continuous advancements in medical imaging, the traditional diagnostic model for fractures is undergoing technological innovations towards intelligence and automation. Consequently, currently, deep-learning-based technologies are gradually being applied in the field of bone injury identification, and they exhibit significant advantages in clinical diagnosis compared with conventional detection methods [8]. In the architecture of deep-learning detection models, there are currently two major categories. Specifically, traditional detection models adopt two-stage models, such as Region with Convolutional Neural Network features (R-CNN) [9] and Faster R-CNN [10]. These models first extract candidate regions and then classify the candidate regions and perform bounding-box regression. Unfortunately, these models generally take a long time, occupy a large amount of disk space, and have a lot of model redundancy. To address such issues, single-stage models have been proposed, such as Single Shot MultiBox Detector (SSD) [11] and the You Only Look Once (YOLO) series [12]. Remarkably, with basically the same detection accuracy, these models treat the object-detection task as a regression problem. By omitting the redundant calculations in steps such as candidate-region extraction, they significantly improve the detection speed.

In recent years, deep learning has emerged as a transformative force in medical imaging fracture detection, offering innovative solutions to enhance diagnostic efficiency and accuracy. Within this rapidly evolving field, the YOLO series of models has garnered significant attention, with multiple studies demonstrating their clinical potential: Son et al. [13] developed a radiologist-independent deep-learning system for mandibular fracture detection in panoramic X-rays, directly validating YOLO’s feasibility for fracture identification tasks. Building on this foundation, Hržić et al. [14] applied the YOLOv4 architecture to wrist fracture diagnosis, introducing the YOLO 512 Anchor model-AI. Critically, their work mitigated fracture missed diagnosis rates while highlighting synergies between AI and clinicians, thereby establishing a pragmatic, child-specific solution for pediatric wrist fracture detection. Concurrently, Ahmed et al. [15] addressed dual challenges in pediatric wrist imaging radiology resource scarcity and inefficiencies of traditional two-stage detection models by exploring single-stage YOLO-based alternatives. Their findings, notably a mean average precision (mAP) of 77% for YOLOv8x, underscored single-stage models’ capacity to advance pediatric wrist fracture diagnostics. Expanding the scope further, Zou et al. [16] proposed a whole-body fracture detection framework using an improved YOLOv7 variant, achieving an mAP of 80.2%. This outcome specifically validated the clinical utility of enhanced loss functions and attention mechanisms in fracture detection workflows. Finally, Zheng et al. [17] leveraged 4493 X-rays from two institutional datasets, employing Demo-YOLO for data processing and augmentation. Their results conclusively demonstrated that dataset augmentation yielded substantial improvements in model detection accuracy.

Overall, in the studies mentioned above, researchers used public datasets or datasets processed via data augmentation, directly applied YOLO-series models or their improved variants, and successfully detected fracture areas in various parts of the body. However, three critical limitations remain: suboptimal mean average precision, unresolved missed detections and localization errors in small fractures, and poor data generalization. To address these issues, this study selected YOLOv11n as the baseline, optimized its localization and precision, and proposed the Bone Fracture-YOLOv11n (BF-YOLOv11n) model for whole-body, multi-site fracture detection. The main contributions are as follows:

(1) First of all, multiple public datasets were collected. Subsequently, a data augmentation technique combining random rotation, translation, flipping, and content recognition filling was proposed. This was performed to effectively alleviate the problem of data scarcity and thereby enhance the generalization ability of the model.
(2) Building on the foundation of Multi-Scale Convolutional Attention (MSCA) [18], a Bone-MSCA attention mechanism module was designed. Specifically, this module combines multi-directional convolution, deformable convolution, edge enhancement, and channel attention mechanism. Then, the Bone-MSCA was integrated into the backbone network of the YOLOv11n model. The purpose of this integration was to accurately extract the features of the fracture area. By enhancing the edges of the fracture lines through edge enhancement and adaptively adjusting the feature channels via channel attention, global information was effectively aggregated. As a result, the perception of spatial details in X-ray fracture images was significantly improved.
(3) Finally, the Focal mechanism [19] was combined with Smoothed Intersection over Union (SIoU) [20] as the loss function. This combination aimed to enhance the sensitivity to small fracture areas and optimize the accuracy of bounding-box regression. Consequently, the accuracy of medical X-ray fracture area detection was further improved.

The rest of this paper is organized as follows. Section 2 introduces the acquisition of medical X-ray fracture images and data augmentation methods, elaborates on the model structure and principle of YOLOv11n, and provides a detailed description of the BF-YOLOv11n model; Section 3 contains detailed information about all the experiments; Section 4 presents the research conclusions and future prospects.

## 2. Materials and Methods

### 2.1. Acquisition of Medical X-Ray Fracture Images

**Figure 1:**
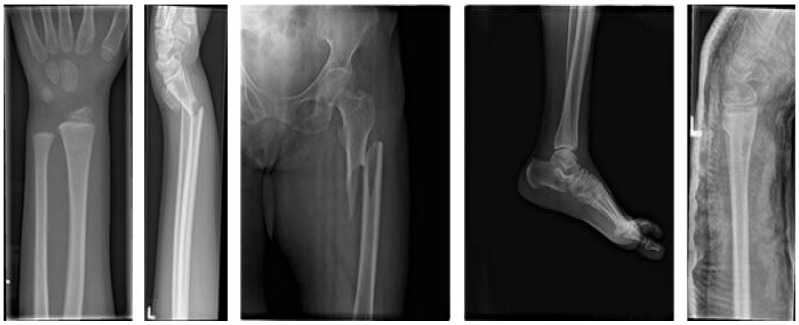
Sample Images from the X-ray Fracture Dataset.

In this research, multiple public datasets of medical X-ray images of bone fractures in various parts of the whole body across all age groups were integrated [21,22,23]. Specifically, a total of 34,990 original images covering all age ranges and various parts of the whole body including limbs and trunk were included. Subsequently, through a multi-level screening process initially postgraduate students majoring in orthopedics eliminated three types of images based on clinical imaging diagnostic standards normal images without fractures blurred images caused by equipment noise motion artifacts or improper exposure and non-standard position images where the proportion of bones outside the field of view exceeds 30% such as excessively tilted limb X-rays. Next, orthopedic and radiology physicians double-blindly excluded cases according to the imaging features of fracture diagnosis in the guidelines of the American Academy of Orthopaedic Surgeons (AAOS) including old fractures with healing period over 6 weeks non-traumatic bone lesions such as bone tumors infections and suspicious cases that cannot be clearly judged due to artifact interference such as areas obscured by metal implants. Eventually, 10,153 valid fracture images were retained. Some image samples are presented in Figure 1.

This study employed a data augmentation method encompassing random rotation, translation, flipping, and core content-aware filling [24] to enhance medical X-ray fracture images. Among these techniques, content-aware filling serves as a crucial technical pillar, playing an irreplaceable role in simulating image diversity caused by variations in shooting angles, equipment, and patient positions. It not only works in conjunction with other transformation methods to improve dataset coverage and model generalization but also ensures the natural integration of fracture regions with surrounding tissues by accurately inferring and filling blank areas generated after image transformations. This maximizes the preservation of image authenticity and effectively avoids artificial noise interference that might be introduced by traditional filling methods. This technical feature enables the YOLOv11 model to more accurately capture the subtle variability of fracture features while learning rotation invariance and positional robustness. Consequently, under limited data conditions, the model’s accuracy in locating complex fractures has been improved. Techniques such as random selection, translation, and flipping can be easily implemented using OpenCV and NumPy libraries. The research also introduces a novel content-aware filling approach, which infers and fills blank areas after image transformations to ensure natural integration with the image content.

#### 2.1.1. Blank Area Detection

Let the image be represented as a three-dimensional tensor *I* ∈ R*^H^*^× *W* × *C*^. Typically, blank areas result from invalid pixels that emerge after affine transformations, including rotation and translation. Specifically, when the transformed pixels fail to map to the valid region of the original image, their values are set to the background color [0, 0, 0]. Subsequently, for every pixel point (*i*,*j*), a binary mask M ∈ {0,1}*^H^*^× *W*^ is created, as described below:

(1) $$M_{i,j}=\left\{\begin{array}{l} 1\;\mathrm{if}\;I_{i,j}=[0,0,0]\;\\ 0\;\mathrm{otherwise} \end{array}\right.$$

#### 2.1.2. Edge Color Sampling and Statistical Inference

Achieving a more natural filling effect demands inferring colors from the edge pixels within the valid region of the image. In fact, since the edge colors of the image are adjacent to the blank areas, it is reasonable to use them as the basis for the filling operation. Then, for each RGB channel, the subsequent formula is employed to calculate the mean value of the edge pixels:

(2) $$Top=\frac{1}{W}\sum_{j=0}^{W-1}I_{0,j,c}$$

(3) $$Bottom=\frac{1}{W}\sum_{j=0}^{W-1}I_{H-1,j,c}$$

(4) $$Left=\frac{1}{H}\sum_{j=0}^{H-1}I_{i,0,c}$$

(5) $$Right=\frac{1}{H}\sum_{j=0}^{H-1}I_{i,W-1,c}$$

where the notations *Top*, *Bottom*, *Left*, and *Right*, respectively*,* denote the upper, lower, left, and right edges of the edge-sampling region.

Subsequently, a global average color operation is carried out:

(6) $$color_{\mathrm{avg}}=\frac{Top+Bottom+Left+Right}{4}$$

By leveraging the continuity of the image gradient, the filled area can be seamlessly integrated with the surrounding pixels through the minimization of an energy function. The Poisson blending formula is as follows:

(7) $$color_{\mathrm{Seamless}}=\underset{I_{\mathrm{fill}}}{\min}{\iint_{\Omega }\left|\nabla I_{\mathrm{fill}}-\nabla I_{\mathrm{src}}\right|}^{2}dxdy$$

where *Ω* denotes the filling region, and ∇ stands for the gradient operator. Specifically, |∇*I*_fill_ − ∇*I*_src_| quantifies the difference between the gradient of the source image and the gradient of the filled image.

#### 2.1.3. Final Filling Operation

During the initial stage, a simple mean-filling approach is adopted to rapidly cover the blank regions. This is performed to circumvent the high computational expenses that would result from directly applying Poisson blending to large-scale blank areas. Specifically, the corresponding formula is as follows:

(8) $$I_{\mathrm{fill}}^{1}[M]=color_{\mathrm{avg}}$$

During the subsequent stage, $I_{\mathrm{fill}}^{1}$ is regarded as the source image *I*_src_. Building upon the mean-filling process, the gradient continuity between the filled region and the neighboring pixels is further enhanced to eradicate color discontinuities. Specifically, the relevant formula is as follows:

(9) $$I_{\mathrm{fill}}^{2}=\underset{I}{\min}{\iint_{\Omega }\left|\nabla I-\nabla I_{\mathrm{fill}}^{1}\right|}^{2}dxdy$$

**Figure 2:**
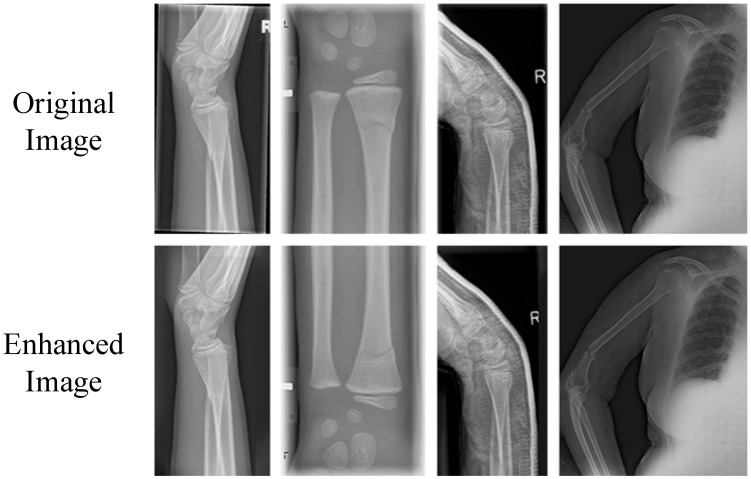
Sample Display of Augmented X-ray Fracture Dataset.

Through coarse filling to offer reasonable initial values and Poisson blending to fine-tune the edges, an equilibrium between efficiency and quality is established. Notably, several of the augmented samples are depicted in Figure 2.

Following data augmentation, the images underwent rigorous screening by orthopedic postgraduate students, orthopedic physicians, and radiologists. Ultimately, 4114 augmented images were selected. These images conformed to the criteria regarding realism and defect features and were then incorporated into the dataset of this research. As a result, the comprehensively constructed medical X-ray fracture dataset encompasses a total of 14,267 images.

By utilizing the dataset annotation tool LabelImg, the bounding-box annotation of 14,267 image samples is manually carried out. Specifically, the category and location information of fracture targets within these images are annotated. The label is designated as “Fracture”. The annotation results are then saved as txt files, thus finalizing the construction of the medical X-ray fracture dataset in the YOLO format. Thereafter, the images in the dataset are randomly partitioned into a training set (9987 images), a validation set (2854 images), and a test set (1426 images) at a ratio of 7:2:1.

### 2.2. Medical X-Ray Fracture Target Detection Model and Its Optimization

#### 2.2.1. YOLOv11n

YOLOv11, introduced by Ultralytics in 2024 [25], is a member of the YOLO series. Its network architecture, built upon YOLOv8, adopts a lightweight object-detection framework. The core elements of this architecture consist of three main parts: the Backbone, the Neck, and the Head. 

In the backbone network, a total of 13 convolutional layers are configured, and three key module upgrades have been implemented compared to previous models such as YOLOv8n: The C3k2 module replaces the C3 module in YOLOv8n, adopting a hybrid convolution kernel of 3 × 3 and 1 × 1, which optimizes information flow through a smaller receptive field, effectively reducing computational overhead while preserving local feature details such as subtle fracture lines; The C2PSA module integrates partial spatial attention mechanism into the C2f structure, replacing the basic C2f module of YOLOv8n, which enhances the model’s attention to key fracture boundaries through adaptive weighted spatial regions and improves the feature extraction accuracy of low-contrast bone fissures. The neck network includes 6 convolutional layers and adopts the PANet structure instead of the traditional FPN, significantly improving small target detection capability through a top-down path aggregation strategy; The head network integrates dynamic head mechanism and depthwise separable convolution technology to achieve adaptive weight adjustment, reducing redundant computation while improving detection accuracy and efficiency; The Concat module in the neck fuses multi-scale features by concatenating feature maps in the channel dimension, enriching feature representation and improving detection accuracy. Overall, these technical improvements enable YOLOv11 to significantly reduce model parameters and computational requirements while maintaining high-performance detection.

The reason for selecting YOLOv11n as the baseline model is based on its comprehensive advantages in lightweight design, real-time performance, and network structure adaptability. Additionally, the C3k2, SPPF, and C2PSA modules in its backbone network can effectively capture subtle features of fracture regions in X-ray images, laying a foundation for the subsequent integration of the Bone-MSCA module and optimization of the Focal-SIoU loss function.

#### 2.2.2. Optimized Model for Medical X-Ray Fracture Target Detection

YOLOv11n, a lightweight object detection model, exhibits certain limitations when applied to medical X-ray fracture detection. Specifically, on one hand, the lightweight nature of the model might result in the oversight of minute fractures or low-contrast bone fissures because of its inadequate feature extraction capabilities. Moreover, it struggles to discern the texture disparities among complex fracture types, such as comminuted fractures. On the other hand, YOLOv11n is highly sensitive to image noise and artifacts. In low-quality medical X-ray images, it is likely to make incorrect judgments. Additionally, the pursuit of real-time performance optimization may come at the cost of positioning accuracy, thereby impeding the accurate delineation of fracture boundaries. In response to the above issues, this research presents an enhanced model. Specifically, it conducts a targeted improvement on the MSCA. In the network architecture, multi-directional convolution, deformable convolution, an edge enhancement module, and channel attention (Squeeze-and-Excitation Networks, SE) [26] are incorporated, giving rise to the design of Bone-MSCA. 

**Figure 3:**
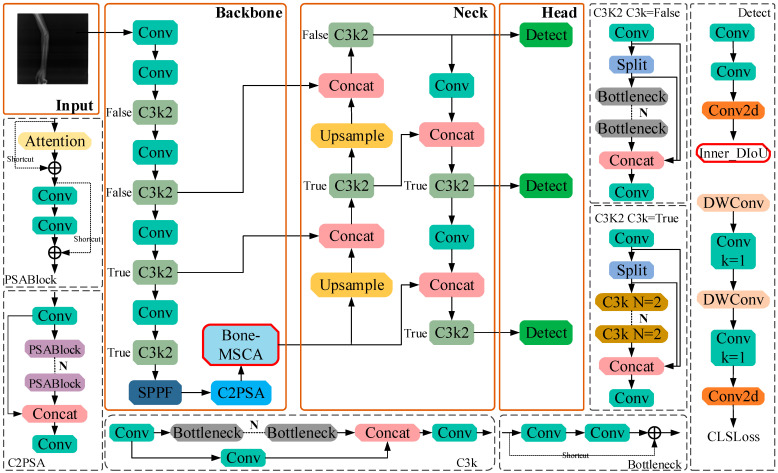
The network structure of BF-YOLOv11n.

Bone-MSCA is then integrated into the terminal part of the YOLOv11n backbone network. By means of multi-directional convolution and deformable convolution, the irregular shapes of fracture lines can be dynamically captured. In combination with Sobel edge enhancement, the high-contrast features of bone cortex interruptions are accentuated. The SE attention mechanism serves to suppress soft-tissue artifact noise, thereby minimizing false detections and missed detections. Simultaneously, lightweight grouped convolution guarantees computational efficiency. To enhance the sensitivity of detecting tiny fractures, the weights of challenging samples are increased. To refine the positioning accuracy of complex fractures, the interference from bone overlap and noise is mitigated. Through category imbalance adjustment, background interference is curbed, and the model’s ability to learn fracture regions is bolstered. Focal-SIoU jointly optimizes bounding-box regression and classification confidence, accelerating the model’s convergence rate and ensuring high robustness in low-quality images. The network structure of the Bone Fracture-YOLOv11n (BF-YOLOv11n) model is illustrated in Figure 3.

#### 2.2.3. BF-MSCA Module

Bone-MSCA harnesses multi-directional convolution to comprehensively capture the characteristics of fracture lines oriented in various directions. Simultaneously, deformable convolution adapts to the irregular geometries of fracture lines, enabling precise localization. The edge enhancement module, leveraging the Sobel kernel, accentuates the edges of fracture lines, thereby enhancing detection accuracy. The channel attention mechanism emphasizes the features of fracture-relevant channels, boosting the model’s performance. Moreover, the residual connection preserves the original information, facilitating the model’s convergence. Furthermore, grouped convolution is employed to curtail parameters and computational intricacy, achieving both computational efficiency and a lightweight architecture. The parameter-sharing strategy inherent in grouped convolution helps prevent overfitting and bolsters the model’s generalization ability. By utilizing convolution kernels of varying sizes and dilation rates, multi-scale feature fusion is realized, which effectively detects target regions of different dimensions. Notably, each module within Bone-MSCA has a well-defined function. This clarity enhances the model’s interpretability, enabling researchers to more easily comprehend the decision-making process and optimize the model accordingly.

**Figure 4:**
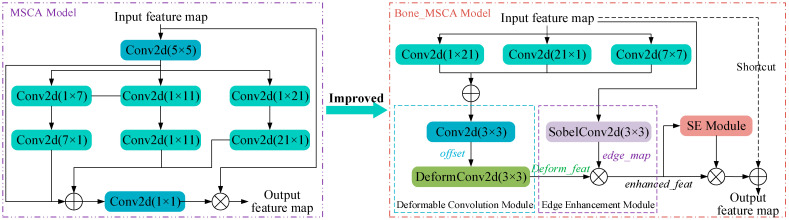
Bone-MSCA Attention Mechanism Structure Diagram.

In this research, Bone-MSCA is integrated into the terminal part of the YOLOV11 backbone network. Specifically, by capitalizing on modules within Bone-MSCA, including multi-directional convolution, deformable convolution, edge enhancement, and channel attention, the multi-directional and irregular shape features of fracture lines can be precisely captured. Concurrently, edge and key channel features are accentuated. Through this approach, Bone-MSCA can effectively mirror the details and crucial features present in medical X-ray fracture images. It optimizes the amalgamation of multi-dimensional features, endowing the network with greater flexibility and accuracy when localizing fracture regions. Moreover, it enhances the robustness of detection in the face of complex fracture scenarios and low-quality images. As a result, the capacity of YOLOV11 to represent fracture-related features is significantly boosted. The structural design of Bone-MSCA is meticulously crafted to suit the characteristics of fracture images. Its implementation in YOLOV11 leads to superior performance in the fracture detection task. The architecture of the Bone-MSCA attention mechanism is depicted in Figure 4.

As is evident from Figure 4, Bone-MSCA initiates with performing vertical convolution, horizontal convolution, oblique dilated convolution, and feature fusion operations on the input feature map X ∈ R^*B* × *H* × *W* × *C*^. Specifically, the corresponding mathematical expression is as follows:

(10) Vert=X∗Conv(1×21)

(11) Hori=X∗Conv(21×1)

(12) $$Diag=X*d_{1}Conv(7\times 7)+X*d_{2}Conv(7\times 7)$$

(13) Fuse=Vert+Hori+Diag

where *Vert*, *Hori*, and *Diag*, respectively*,* denote vertical, horizontal, and oblique dilated convolutions. Meanwhile, Fuse stands for feature fusion, and di indicates the dilation rate.

The mathematical formulation for the deformable convolution module is presented as follows:

(14) $$offset=Conv_{3\times 3}(Fuse)$$

(15) deform_feat=DeformConv(Fuse,offset)

where *offest* represents the offset amount, and *offest* ∈ R^*B* × *H* × *W* × (2 × 3 × 3 × *C*)^; DeformConv represents the deformable convolution operation.

The mathematical representation of the edge enhancement module is given as follows:

(16) $$edge\_map=\sigma (X*W_{\mathrm{sobel}})$$

(17) enhanced_feat=deform_feat⊗(1+edge_map)

where σ denotes the Sigmoid function. Meanwhile, *W*_sobel_ represents the fixed Sobel kernel, with *W*_sobel_ ∈ R^3 × 3 × *C*^.

The mathematical expression of the channel attention enhancement module is:

(18) $$\left\{\begin{array}{ll} z=GlobalAvgPool(enhanced\_feat) & \\ s_{1}=W_{1}\times z & W_{1}\in R^{C/(4\times C)}\\ s_{2}=ReLU(s_{1}) & \\ s=\sigma (W_{2}\times s_{2}) & W_{2}\in R^{(C\times C)/4} \end{array}\right.$$

(19) refined_feat=enhanced_feat⊗s

where *GlobalAvgPool* and *ReLU* represent the global average pooling operation and the ReLU activation function, respectively; *W_i_* is the weight of the fully connected layer of the SE module; the final channel weight *s* ∈ R^*B* × 1 × 1 × *C*^; after channel recalibration, the *refined-feat* is obtained.

Ultimately, the output feature map is derived via the residual connection. The corresponding formula is as follows:

(20) output=X+refined_feat

The Bone-MSCA attention mechanism integrates multi-directional convolution, deformable convolution, edge enhancement, and channel attention modules to comprehensively extract features across diverse directions and scales. Specifically, it discerns the orientation of fracture lines via multi-directional convolution, adapts to irregular shapes through deformable convolution, accentuates edges using edge enhancement, and modulates feature channels with channel attention. Collectively, these components effectively enhance the spatial detail perception of medical X-ray fracture images. When combined with the YOLOv11n backbone network, this mechanism further mitigates background interference, thereby bolstering detection capability across a broad spectrum of fracture scenarios including subtle fracture lines and intricate fracture morphologies. Ultimately, it provides a robust and precise feature representation for medical X-ray fracture detection.

#### 2.2.4. Focal-SIoU Loss Function

By default, YOLO v11n adopts the Complete Intersection over Union (CIoU) loss function as the reference approach for bounding-box positioning. In the calculation process, this loss function incorporates a regression term related to the aspect ratio of the bounding box. However, it suffers from two primary drawbacks. Specifically, it does not efficiently handle the imbalance issue between positive and negative samples. Moreover, it imposes insufficient constraints on the spatial orientation features of the detection boxes, a phenomenon that becomes especially prominent when the length-width characteristics of the target objects are not distinct.

In response to the aforementioned issues, a two-pronged improvement strategy is proposed in this research. Specifically, on one hand, the original loss calculation approach is substituted by the SIoU function with integrated angular constraints, which enhances the directional sensitivity of the detection boxes. On the other hand, the introduction of the Focal weight adjustment mechanism aims to mitigate the impact of category distribution imbalance during the training phase, particularly in the common scenario within medical imaging where the quantity of healthy area samples far exceeds that of lesion areas. In the domain of object detection, the IOU, a crucial quantitative metric for detection accuracy, is defined as the ratio of the overlapping area between the predicted region and the ground-truth annotated region to the area of their union, as presented in Equation (21):

(21) $$IoU=\frac{\left|B\cap B_{T}\right|}{\left|B\cup B_{T}\right|}$$

where ***B*** and ***B***_T_ respectively denote the predicted bounding-box region and the actual bounding-box region.

**Figure 5:**
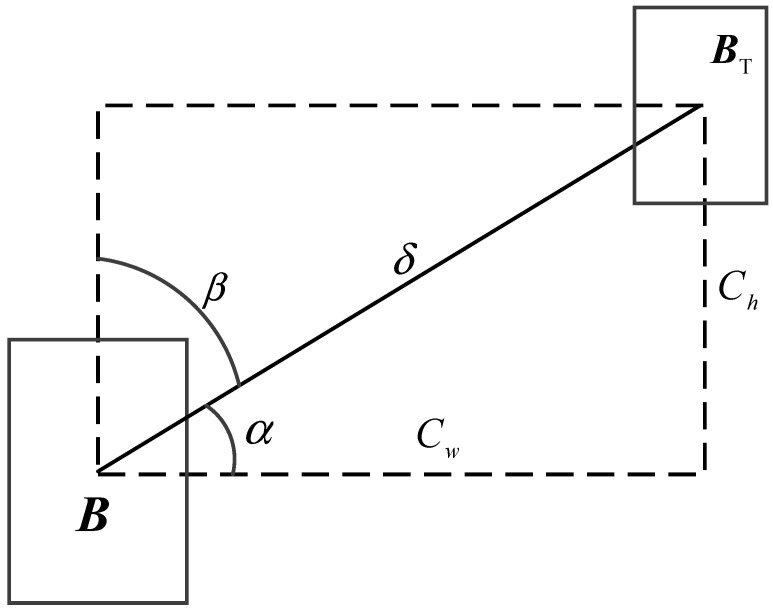
Schematic diagram of angle cost calculation in SIoU.

The SIoU loss function innovatively incorporates four crucial cost functions, namely the angle, distance, shape, and intersection-over-union functions, via a multi-dimensional geometric constraint mechanism. Specifically, a schematic illustration depicting the calculation of the angle cost is presented in Figure 5.

The angle cost function serves to capture angle information. The corresponding formula is presented as follows:

(22) $$\Lambda =1-2\sin^{2}(\arcsin(x)-\frac{\pi }{4})$$

where

(23) $$x=c_{h}/\delta =\sin(\alpha )$$

(24) $$\sigma =\sqrt{{\left(b_{\mathrm{cx}}^{\mathrm{gt}}-b_{\mathrm{cx}}\right)}^{2}-(b_{\mathrm{cy}}^{\mathrm{gt}}-b_{\mathrm{cy}})}$$

(25) $$c_{h}=\max(b_{\mathrm{cy}}^{\mathrm{gt}},b_{\mathrm{cy}})-\min(b_{\mathrm{cy}}^{\mathrm{gt}},b_{\mathrm{cy}})$$

The position cost changes with the angle cost, as shown in Equation (26):

(26) $$\Delta =\sum_{t=x,y}\left(1-e^{-\gamma \rho_{t}}\right)$$

where

(27) $$\rho_{x}=(\frac{b_{\mathrm{cx}}^{\mathrm{gt}}-b_{\mathrm{cx}}}{c_{w}}),\;\rho_{y}=(\frac{b_{\mathrm{cy}}^{\mathrm{gt}}-b_{\mathrm{cy}}}{c_{h}}),\;\gamma =2-\Lambda$$

where ($b_{\mathrm{cx}}^{\mathrm{gt}}$, $b_{\mathrm{cy}}^{\mathrm{gt}}$) and (*b*_cx_, *b*_cy_) respectively denote the center point of the actual bounding box and the center point of the predicted bounding box.

The shape cost is calculated based on the distance cost, as depicted in Equation (28):

(28) $$\Omega =\sum_{t=w,h}{\left(1-e^{-\varpi_{t}}\right)}^{\theta }$$

where

(29) $$\varpi_{w}=\frac{\left|w-w^{\mathrm{gt}}\right|}{\max(w,w^{\mathrm{gt}})},\varpi_{h}=\frac{\left|h-h^{\mathrm{gt}}\right|}{\max(h,h^{\mathrm{gt}})}$$

where the notations *w*^gt^ and *h*^gt^ respectively denote the width and height of the actual bounding box, and *θ* is set to 1.

In summary, the formula for calculating the overall SIoU loss function is presented as follows:

(30) $$L_{\mathrm{SIoU}}=1-IoU+\frac{\Delta +\Omega }{2}$$

By balancing the evaluation of the importance of high-and low-confidence samples, this research integrates the Focal mechanism with SIoU. Specifically, through the introduction of a balancing factor *τ*, the Focal mechanism adjusts the sample weights, allowing the model to focus more intently on hard-to-distinguish samples. The value of τ can be adjusted flexibly according to the real-world situation to achieve optimal results. The relevant formula is presented as follows:

(31) $$L_{\text{Focal-SIoU}}=-{\left(1-IoU\right)}^{\tau }\log(IoU)L_{\mathrm{SIoU}}$$

The Focal-SIoU Loss function, shaped by the two mechanisms, dynamically regulates the weight equilibrium between healthy tissues and fracture lesions via the gradient-focusing mechanism. This effectively mitigates the sample-imbalance issue in cases where the proportion of the fracture area is minuscule. When integrated with the direction-morphology dual-constraint model, the function boosts the positioning precision of minute or inclined fractures, thereby ultimately enhancing the overall detection performance of the model.

#### 2.2.5. Training Environment and Methods

The training environment for this research is built upon the Pytorch framework. It serves in the establishment, training, and analysis of the medical X-ray fracture detection model. Specifically, the detailed configurations of the software and hardware platforms are presented in Table 1.

During the training stage, the hyperparameters are configured as follows. First, the images are uniformly resized to 640 × 640 pixels. Subsequently, the batch size is set to 8, and the number of threads is configured to 4. The Stochastic Gradient Descent (SGD) model is chosen as the optimizer. Then, a full 200-iteration training process is implemented. Regarding the learning rate, it is adjusted according to the cosine annealing decay strategy [27]. Meanwhile, other hyperparameters maintain the default values provided in the YOLOv11 source code.

In order to assess the performance of the medical X-ray fracture target detection model, this research utilizes the following metrics to analyze the experimental results: Precision (*p*), Recall (*R*), Average Precision (*AP*), F1-Score (*F*_1_), mean Average Precision (mAP), Parameters, GFLOPs, as well as Inference metrics including preprocessing, inference, and post-processing stages [28]. Specifically, the mathematical expressions are as follows:

(32) $$P=\frac{T_{p}}{T_{p}+F_{p}}\times 100\%$$

(33) $$R=\frac{T_{p}}{T_{p}+F_{n}}\times 100\%$$

(34) $$F_{1}=2\times \frac{P\times R}{P+R}$$

(35) $$mAP=\frac{\sum_{i=1}^{N}P_{i}(R)dR}{N}\times 100\%$$

where *T_p_* denotes the number of samples with correctly detected defects. Similarly, *F_p_* denotes the number of samples mis-identified as having defects, and *F_n_* represents the number of samples where defects are not accurately detected. The symbol *n* represents the number of defect categories. In the context of this research, since the focus is uniformly on detecting the fracture area, *n* = 1.

## 3. Results

### 3.1. Impact of Dataset Expansion on Model Performance

**Figure 6:**
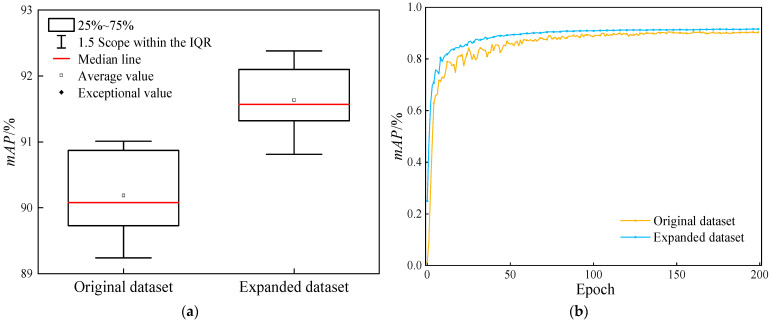
Comparison of the detection performance of the YOLOv11n model before and after dataset augmentation. (**a**) Comparison chart of box plots for the mAP50 of models; (**b**) Comparison chart of mAP50 changes.

In order to objectively assess the influence of the augmented dataset presented in this research on the model, the precision boxplot and the mAP50 (*IoU* = 0.5) variation graph are utilized to contrast the detection performance of YOLOv11n for the medical X-ray fracture dataset before and after augmentation. Specifically, the relevant data is presented in Figure 6.

As is manifest from Figure 6, the comparative analysis of data before and after augmentation reveals that the utilization of the augmented dataset leads to a significant enhancement of the model’s performance during YOLOv11 training. Box-plot analysis demonstrates that the median of the augmented dataset exceeds that of the original dataset. Although the distribution ranges of the 25–75% quantiles are comparable, the overall performance center experiences an upward shift. This indicates that the augmentation strategy effectively boosts the average detection accuracy of the model. Throughout the training process, the mAP50 value of the augmented dataset consistently remains higher than that of the original dataset. Specifically, at the final training endpoint, the mAP50 value increases from 90.19% to 91.64%, and during long-term training, the mAP50 value shows a tendency towards stability. Consequently, a rational data augmentation strategy can improve the model’s training outcomes and performance.

### 3.2. Performance Comparison of Different Attention Mechanisms

**Figure 7:**
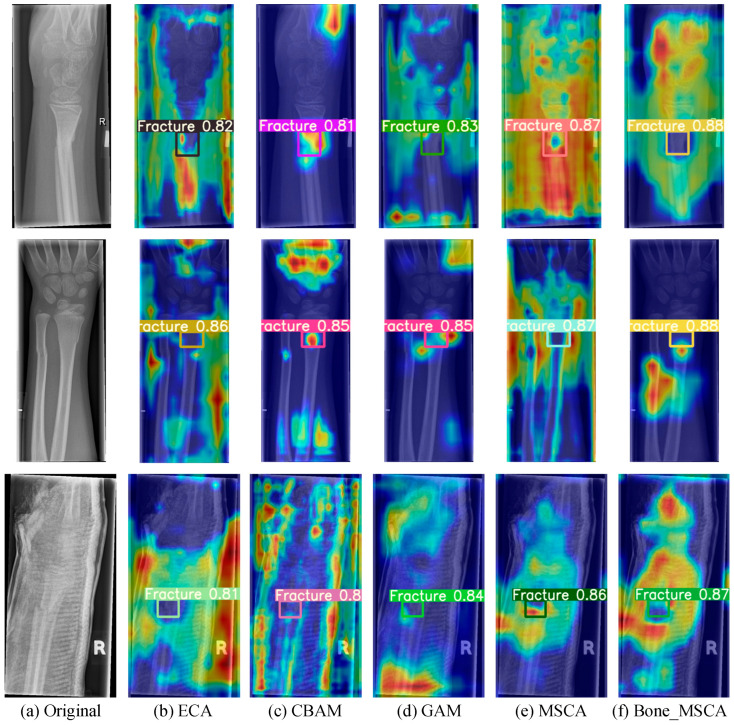
Visualization Analysis of Heatmaps for Model Detection Results under Different Attention Mechanisms. (**a**) Original image; (**b**) Using ECA attention mechanism; (**c**) Using CBAM attention mechanism; (**d**) Using GAM attention mechanism; (**e**) Using MSCA attention mechanism; (**f**) Using Bone_MSCA attention mechanism. In the figure, blue indicates low intensity, yellow indicates medium intensity, and red indicates high intensity. This color coding is used to visually distinguish the fracture detection sensitivity of different algorithms.

By accurately assessing the function of the attention mechanism in boosting the detection performance of medical X-ray fracture areas, this research undertakes comparative experiments with multiple attention mechanisms, namely Efficient Channel Attention (ECA) [29], Convolutional Block Attention Module (CBAM) [30], Global Attention Mechanism (GAM) [31], MSCA, and Bone-MSCA. During training, Gradient-weighted Class Activation Mapping (Grad-CAM) [32] is employed to generate a heatmap for a particular fracture in each instance. This serves to visually illustrate how the model learns feature information when handling different defect targets. By analyzing the brightness variations in specific regions of the heatmap, the areas within the image that significantly influence the model’s output can be uncovered. Figure 7 presents a comparison of heatmaps obtained using different attention mechanisms.

As can be clearly observed from Figure 7, upon the completion of training, the Bone-MSCA attention mechanism put forward in this research can detect medical X-ray fracture regions with higher confidence levels and more vivid regional colors. Moreover, it devotes more attention to defects. The Bone-MSCA realizes cross-scale defect representation via multi-branch parallel convolution. Additionally, it introduces an adaptive weight-fusion mechanism. This mechanism adaptively adjusts the contribution ratio of multi-scale features, enhances the separation between defects and the background, and improves the fine-grained detection capability for multi-morphological surface defects under complex lighting conditions.

### 3.3. Comparative Experiment of Loss Functions

In order to validate the influence of various loss functions on the model’s performance, comparative experiments are conducted. These experiments utilize the CIoU, Focal-IoU, SIoU, Focal-CIoU, and Focal-SIoU loss functions, building upon the original improved model. Specifically, the experimental results are presented in Table 2.

As is evident from Table 2, when compared to other loss functions, the Focal-SIoU loss function employed in this research exhibits the best F1 and mAP50 values, reaching 88.86% and 92.38%, respectively. This is because SIoU enhances the model’s positioning precision for irregular fracture areas through the introduction of an angle-penalty mechanism. Meanwhile, Focal Loss boosts the model’s sensitivity to small-target and low-contrast fractures by suppressing the weight of the background area, thus significantly improving the precision. The combined effect of these two functions not only mitigates the issue of highly unbalanced positive and negative samples in medical images but also strikes a balance between precision and recall, thereby achieving a more robust detection performance.

### 3.4. Performance Comparison of BF-YOLOv11n Ablation Experiments

In this research, ablation experiments are employed to validate the viability of the strategy that incorporates the Bone-MSCA attention mechanism and the Focal-SIoU loss function into the YOLOv11n model. Specifically, the experimental results are presented in Table 3.

It is clear from Table 3 that within the medical X-ray fracture detection task, incorporating Bone-MSCA and Focal-SIoU significantly enhances the model’s performance. Bone-MSCA integrates multi-scale fracture features via oblique dilated convolution to capture directional fracture line disparities and, in conjunction with the SE module to suppress bone-overlap noise, intensifies the response of key regions. This leads to an increase in precision from 89.23% to 93.56% and a 1.24% elevation in mAP50 a core metric for detection accuracy. Conversely, Focal-SIoU mitigates the sparsity issue of fracture regions in X-ray images by dynamically adjusting the weights of easy- and hard-to-detect samples via the Focal mechanism to enhance blurred fracture detection and optimizing the direction-aware bounding box. While preserving the model’s lightweight characteristic, it boosts the recall by 0.92% a metric reflecting the model’s ability to capture all true fractures.

When these two components collaborate, the feature-extraction capacity of Bone-MSCA and the loss-guiding function of Focal-SIoU create a closed-loop. As a result, the comprehensive metric *F*_1_ balancing precision and recall experiences a 2.52% increase, indicating robust optimization of overall detection performance.

### 3.5. Comparative Experiments of Mainstream Object Detection Models

In order to validate the efficacy of the BF-YOLOv11n model put forward in this research, comparative experiments are carried out. These experiments involve the mainstream single-stage object detection model SSD and the recent nano-level versions of the YOLO series, namely YOLOv8n, YOLOv9t [33], YOLOv10n, YOLOv11n [34], YOLOv11n + ResNet_GAM [35], and YOLOv12n [36]. The experimental results are presented in Table 4.

Based on the analysis of the results in Table 4, regarding detection performance, the F1 value of the BF-YOLOv11n model is 50.18%, 16.8%, 22.76%, 2.68%, 2.52%, 0.25%, and 12.99% higher than those of the other six models, respectively. Similarly, its mAP50 value is 33.07%, 1.33%, 0.99%, 1.83%, 1.24%, 0.35%, and 1.46% higher, respectively. Evidently, the BF-YOLOv11n model demonstrates top-tier performance among these models. The recall rate of the SSD model stands at 26.35%, suggesting significant missed detections, thus restricting its practical utility. Additionally, YOLOv11n + ResNet_GAM has a large number of parameters, which is unfavorable for model lightweighting. For YOLOv8n, YOLOv9t, and YOLOv12n, although their recall rates surpass 90%, their precision rates are subpar. Consequently, their F1-Scores are all below 80%, indicating a high false-positive issue. All YOLO-series models exhibit an mAP50 exceeding 91%, signifying their robust positioning capabilities. However, the precision rate fluctuates by 40.67%, underscoring the importance of enhancing feature discrimination and small-target positioning judgment in model optimization. The Parameters and GFLOPs of BF-YOLOv11n are 2.7 × 10^6^ and 6.4 × 10^9^, respectively, which are slightly higher than those of YOLOv11n and YOLOv12n overall. However, the inference time for processing an image is 2.2 ms, which also meets the real-time requirements. Overall, when compared to other models, the BF-YOLOv11n model offers the advantages of precise detection and high robustness in the medical X-ray fracture detection task.

**Figure 8:**
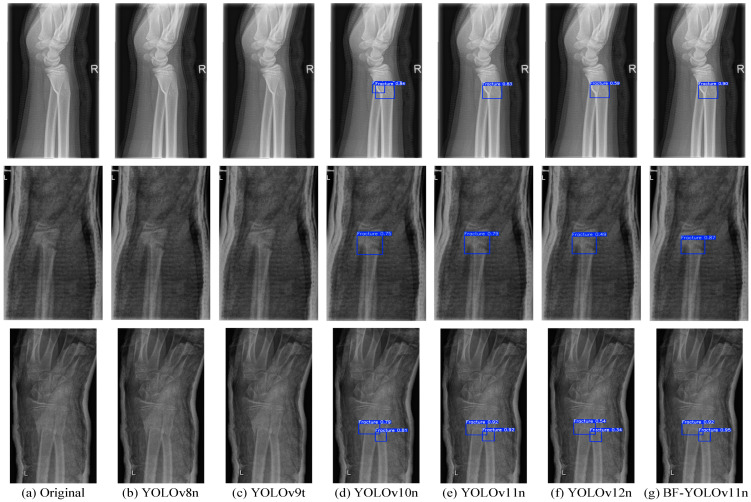
Comparative Effectiveness of Mainstream X-ray Fracture Detection Models. (**a**) Untreated original image; (**b**) Image detection using YOLOv8n; (**c**) Image detection using YOLOv9t; (**d**) Image detection using YOLOv10n; (**e**) Image detection using YOLOv11n; (**f**) Image detection using YOLOv12n; (**g**) Image detection using BF-YOLOv11n.

Given that the mAP50 value of the SSD model is below 60% and does not meet the criteria for precise medical X-ray fracture detection, subsequent reasoning and detection for this model will not be conducted. To evaluate the performance of the BF-YOLOv11n model in detecting medical X-ray fractures, this research performs comparative experimental analyses on YOLOv8n, YOLOv9t, YOLOv10n, YOLOv11n, YOLOv12n, and BF-YOLOv11n, using four groups of images from the test set. Specifically, the related results are presented in Figure 8.

As is evident from Figure 8, owing to the high false-positive issue in YOLOv8n and YOLOv9t, these two models failed to detect any fracture regions. Although YOLOv12n is capable of detecting fracture regions, its positioning capability is inadequate, resulting in generally low detection confidence levels. For YOLOv10n, when it comes to detecting the first image, obvious false detections occur. Both YOLOv11n and BF-YOLOv11n demonstrate good performance in the medical X-ray fracture detection task. Specifically, BF-YOLOv11n exhibits higher detection confidence, more precise target positioning, and greater model robustness, with no instances of missed or false detections.

## 4. Conclusions

This research aims to tackle problems like the occlusion of fine fracture lines, the challenge of identifying early-stage callus, and the susceptibility of traditional models to noise in medical X-ray fracture detection. It does so by putting forward an enhanced BF-YOLOv11n model. First, a dataset expansion strategy is introduced. This strategy encompasses random rotation, translation, flipping, along with an innovative content recognition filling technique. In doing so, it mimics the image diversity in actual clinical scenarios, effectively broadening the dataset’s coverage and bolstering the model’s generalization capabilities. Second, the Bone-MSCA attention mechanism is incorporated. When combined with multi-directional convolution, deformable convolution, an edge enhancement module, and a channel attention mechanism, it enables the effective capture of the irregular morphology and edge features of fracture lines. Simultaneously, it suppresses image noise and artifact interference. Finally, the Focal-SIoU loss function is employed. Through the dynamic adjustment of the weights for easy-to-detect and hard-to-detect samples, along with direction-aware optimization, the imbalance issue of positive and negative samples in medical images is significantly mitigated.

Experimental results indicate that the augmented dataset boosts the model’s mAP50 from 90.19% to 91.64%, validating the positive impact of data augmentation on the detection of complex fracture regions. The precision, recall, F1-Score, and mAP50 of the improved model on the test set, respectively, reach 93.56%, 86.29%, 89.78%, and 92.88%. Evidently, its comprehensive performance outperforms that of other mainstream single-stage detection models. BF-YOLOv11n integrates high precision and strong robustness, offering reliable technical support for clinical fracture-assisted diagnosis.

In future research, efforts will focus on validating the model’s generalizability across broader public datasets and multi-institutional clinical data to enhance rehabilitation outcome prediction capabilities. We will further explore the classification of specific fracture types such as transverse, oblique and comminuted fractures by incorporating anatomical location and morphological features, aiming to develop a subtype-specific detection framework that supports precise clinical decision-making. The application of transfer learning to non-traumatic fractures such as osteoporotic fractures will be explored to develop personalized rehabilitation pathway models. To address clinical needs for real-time rehabilitation monitoring and adaptive treatment planning, we will further reduce model parameters and optimize computational efficiency, enabling deployment on mobile/edge devices for continuous point-of-care rehabilitation assessment.

## Figures and Tables

**Table 1:** Hardware and Software Platform Configuration.

Name	Version Information
System	Ubuntu 22.04.3 LTS
Operating System Kernel	GNU/Linux 5.15.0-124-generic
Central Processing Unit	Intel^®^ Xeon^®^ Platinum 8365A CPU
Graphics Processing Unit	NVIDIA GeForce RTX 3090 24 GB
Python	3.11.10
Deep-learning Frameworks and Libraries	Pytorch 2.4.0, Cuda 12.1.1, cuDNN 9.1.0

**Table 2:** Comparative Experiments on Loss Functions.

Loss Functions	*p*/%	*R*/%	*F*_1_	mAP50/%
CIoU	89.23	85.37	87.26	91.64
Focal-IoU	90.10	86.29	88.15	92.08
SIoU	80.33	92.26	85.88	91.83
Focal-CIoU	90.75	86.44	88.54	92.22
Focal-SIoU	92.08	85.86	88.86	92.38

**Table 3:** Comparison of Ablation Experimental Performance.

Bone-MSCA	Focal-SIoU	*p*/%	*R*/%	*F*_1_	mAP50/%
/	/	89.23	85.37	87.26	91.64
√	/	93.19	85.26	89.05	92.51
/	√	92.08	85.86	88.86	92.38
√	√	93.56	86.29	89.78	92.88

**Table 4:** Comparative Experimental Evaluation of Mainstream Detection Models.

Detection Models	*p*/%	*R*/%	*F*_1_	*mAP50*/%	*Parameters*/*10^6^*	*GFLOPs*/*10^9^*	*Inference*/*ms*
SSD	79.68	26.35	39.60	59.81	26.28	62.7	28.40
YOLOv8n	60.75	91.37	72.98	91.55	3.01	8.1	3.5
YOLOv9t	52.66	92.15	67.02	91.89	1.97	7.6	3.3
YOLOv10n	90.33	84.09	87.10	91.05	2.71	8.2	2.4
YOLOv11n	89.23	85.37	87.26	91.64	2.58	6.3	1.8
YOLOv11n + ResNet_GAM	93.47	85.90	89.53	92.53	5.39	15.2	4.3
YOLOv12n	74.95	90.02	81.80	91.42	2.56	6.3	2.1
BF-YOLOv11n	93.56	86.29	89.78	92.88	2.70	6.4	2.2

## Data Availability

All data used in this study are derived from public datasets, and the corresponding links have been provided in the paper for readers to access.
